# Vagus Nerve Stimulation Attenuates Acute Skeletal Muscle Injury Induced by Ischemia-Reperfusion in Rats

**DOI:** 10.1155/2019/9208949

**Published:** 2019-02-28

**Authors:** Yifeng Zhang, Hewei Li, Menglong Wang, Guannan Meng, Zhenya Wang, Jielin Deng, Meng Wang, Qianqian Zhang, Shengli Yang, Hong Jiang

**Affiliations:** ^1^Department of Cardiology, Renmin Hospital of Wuhan University, Wuhan 430060, China; ^2^Cardiovascular Research Institute, Wuhan University, Wuhan 430060, China; ^3^Hubei Key Laboratory of Cardiology, Wuhan 430060, China; ^4^Department of Orthopedics, Liyuan Hospital, Tongji Medical College, Huazhong University of Science and Technology, Wuhan 430077, China; ^5^Cancer Center, Union Hospital, Tongji Medical College, Huazhong University of Science and Technology, Wuhan 430022, China

## Abstract

Vagus nerve stimulation (VNS) has been shown to attenuate ischemia-reperfusion (I/R) injury in multiple organs. The present study aimed at investigating whether VNS could exert protective effects against I/R injury in the skeletal muscle. Male Sprague-Dawley rats were randomly divided into 3 groups: the control, I/R, and I/R+VNS groups. The skeletal muscle I/R (SMI/R) model was induced by occlusion of the left femoral artery for 2.5 hours followed by reperfusion for 2 hours. The vagal nerve trunk was separated, and VNS was performed during the whole I/R process. The intensity of VNS was optimized in each rat to obtain a 10% reduction in the heart rate relative to the value before stimulation. After the experiment, the blood sample and left gastrocnemius muscle tissues were collected for histological examination, biochemical analysis, and molecular biological detection. During the I/R process, VNS significantly reduced cellular apoptosis, necrosis, and inflammatory cell infiltration compared to sham VNS. The VNS treatment also decreased the inflammatory response, alleviated oxidative stress, and improved vascular endothelial function (*p* < 0.05 for each). In contrast, the I/R group showed an opposite effect compared to the control group. The present study indicated that VNS could protect against SMI/R injury by suppressing excessive inflammation, alleviating oxidative stress, and preserving vascular endothelial function.

## 1. Introduction

Skeletal muscle ischemia-reperfusion (SMI/R) injury is a common disease in clinical practice. It often influences the function of the skeletal muscle and can even be life-threatening [1]. However, there are few effective treatments for SMI/R [2]. Thus, novel effective therapies are needed to prevent SMI/R injury. Numerous studies have demonstrated that the pathogenesis of SMI/R injury is associated with inflammation responses and oxidative stress [3]. Vagus nerve stimulation (VNS) has been shown to exert anti-inflammatory and antioxidative effects [4–6]. Most recently, VNS has been demonstrated to improve I/R injury in multiple organs, including the heart, brain, and kidney [7–9]. VNS applied during the I/R process could reduce the infarct area and improve the prognosis. More importantly, our clinical study has shown that transcutaneous VNS can markedly attenuate myocardial I/R injury in acute myocardial infarction patients undergoing primary percutaneous coronary intervention [10]. However, the protective effects of VNS against SMI/R injury remain unknown. In the present study, using an acute SMI/R model in rats, we aimed to demonstrate the protective effects of VNS against SMI/R injury and further explore the potential mechanisms.

## 2. Materials and Methods

### 2.1. Animals and Experimental Groups

Healthy male Sprague-Dawley rats (250-300 g) were included in this study, and all animals were supplied by the Experimental Animal Center of Renmin Hospital of Wuhan University. The study conformed to the Guidelines for the Care and Use of Laboratory Animals published by the US National Institutes of Health (NIH Publication, revised 1996). All animal studies were reviewed and approved by the Renmin Hospital of Wuhan University Animal Care and Use Committee (ethics clearance number was WDRM. 20180308).

**Figure 1:**
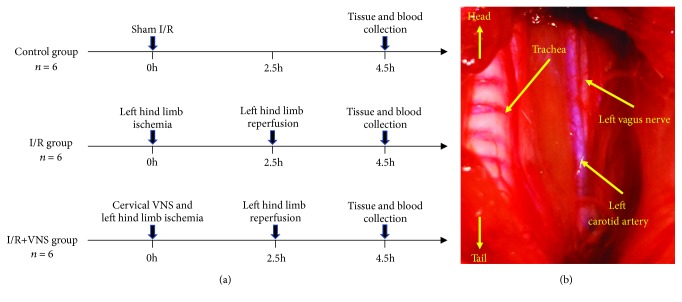
Experimental protocol (a) and location of the vagus nerve (b). I/R: ischemia-reperfusion; VNS: vagus nerve stimulation.

Rats were randomized into 3 groups and received the following treatments: sham operation (control group, *n* = 6), SMI/R with sham VNS (I/R group, *n* = 6), and SMI/R with VNS (I/R+VNS group, *n* = 6). Detailed study protocol is summarized in Figure 1(a).

### 2.2. Acute SMI/R Model

Rats were anesthetized with 2% pentobarbital sodium (40 mg/kg body weight) by intraperitoneal injection. Surface electrocardiography was performed with a PowerLab data acquisition system (8/35, ADInstruments, Bella Vista, Australia). The left femoral artery was exposed by blunt dissection and a pair of atraumatic microvascular clamps were placed (in the control group, only femoral artery exposure was performed). The presence of pulsation under the clamp was checked. After 2.5 h of ischemia, the microvascular clamps were removed and the left hind limb received 2 h of reperfusion as previously described [11].

### 2.3. VNS

The left cervical vagal trunk was isolated as a stimulating target (see Figure 1(b)). Continuous stimulation (20 Hz, 0.1 ms in duration, square waves) was delivered by a stimulator (S20, Jinjiang, Chengdu City, China) through a pair of Teflon-coated silver hooks (0.1 mm in diameter) on the cervical vagal trunk. The stimulation level was defined as the voltage level sufficient to slow the sinus rate or atrioventricular (AV) conduction at 10%, as previously described [12]. The VNS threshold was determined once again prior to each hour of stimulation.

### 2.4. Blood and Tissue Sample Collection

After the entire experimental progress, blood samples were collected from the inferior vena cava. Serum was collected by centrifugation at 3,000 rpm, for 15 min. Tissue specimens were collected from the first half of the left gastrocnemius muscle venter. Each tissue specimen was separated into three parts. The major part (about 5 × 5 × 10 mm^3^) was used for histological analysis. The two minor parts (about 2 × 2 × 2 mm^3^) were used for biochemical and molecular biological analysis. All blood and tissue samples for biochemical and molecular biological analysis were stored at -80°C until use.

### 2.5. Histological Examination

The skeletal muscle tissue samples were submerged in 10% paraformaldehyde solution for 48 h, dehydrated sequentially in an ascending gradient of ethanol, and rinsed in xylene. Then, the tissues were embedded in paraffin. Sections of 5 *μ*m thick were stained with hematoxylin and eosin (H&E) and then examined under a light microscope.

The muscle injury area was estimated in each gastrocnemius muscle section under a microscope according to Yin et al. [13].

Terminal deoxynucleotidyl transferase dUTP nick end labeling (TUNEL) staining was performed with a commercially available kit (Roche Biochemicals, Mannheim, Germany) according to the manufacturer's protocol. Tissue sections were deparaffinized and then hydrated with ethanol. After hydration, the sections were treated with protease K, rinsed with phosphate-buffered saline (PBS), and then incubated with TUNEL reaction reagents. After washing with PBS, the sections were treated with 4′,6-diamidino-2-phenylindole (DAPI) and incubated in a dark environment at room temperature. Stained sections were analyzed using a fluorescence microscope (Nikon DS-U3, Japan). Cells with nuclei containing irregular green particles were defined as TUNEL-positive cells. Cell death was viewed and detected in three random fields of each muscle section and averaged. The TUNEL-positive cell ratio was recorded.

### 2.6. Analysis of Serum Creatine Kinase (CK) and Lactate Dehydrogenase (LDH) in Serum Levels

Serum CK and LDH levels, which reflect skeletal muscle injury severity, were assessed using commercial kits (Changchun Huili Biotech Co. Ltd. China) according to the manufacturer's protocol.

### 2.7. Measurement of Malondialdehyde (MDA), Myeloperoxidase (MPO), and Superoxide Dismutase (SOD) Content Levels in Serum and Tissues

Tissue and serum samples from the control, I/R, and I/R+VNS groups were homogenized. The tissue MPO activity level, the tissue and serum MDA concentration, and SOD activity levels were assayed using commercially available kits (Nanjing Jiancheng Bioengineering Institute, Nanjing City, China) according to the manufacturer's protocol. The samples after reaction were detected by a spectrophotometer. The maximum absorbance values determined for MDA, MPO, and SOD were 532 nm, 460 nm, and 550 nm, respectively.

### 2.8. Western Blot Analysis

Frozen skeletal muscle tissues were lysed with a RIPA lysis buffer (Aspen Biotechnology, Wuhan, China), supplemented with phenylmethanesulfonyl fluoride (Aspen). After homogenization, the supernatant was collected for protein concentration determination using a BCA protein assay kit (Aspen). Equal amounts of protein solution from the homogenates were subjected to SDS-PAGE and then transferred onto a nitrocellulose membrane. After the membrane was blocked with 5% fat-free milk, it was incubated with rabbit anti-endothelial nitric oxide synthase antibody (eNOS, 1 : 1000 dilution, Abcam, Cambridge, UK), rabbit anti-intercellular adhesion molecule 1-antibody (ICAM-1, 1 : 500 dilution, Biorbyt, UK), rabbit anti-vascular cell adhesion molecule-1 antibody (VCAM-1, 1 : 1000 dilution, Abcam, Cambridge, UK), rabbit anti-interleukin 1 beta antibody (IL-1*β*, 1 : 1000 dilution, Abcam, Cambridge, UK), rabbit anti-IL-6 antibody (1 : 1000 dilution, Affinity, San Francisco, USA), and rabbit anti-tumor necrosis factor alpha antibody (TNF-*α*, 1 : 1000 dilution, Proteintech Group, Inc. Wuhan, China). After washing three times with Tris-buffered saline containing Tween (TBST), the membranes were incubated with HRP-goat anti-rabbit antibody (Aspen) at room temperature for 30 min and then washed four times with TBST. The protein bands were visualized by the chemiluminescence method and quantified using analytical software (AlphaEase FC, USA). The relative expression of target proteins was normalized to *β*-actin from the same sample, and the data was normalized by the mean value of the control group.

### 2.9. qRT-PCR

Total RNA was extracted from the frozen skeletal muscle tissues using a Trizol reagent (Invitrogen Life Technologies) according to the manufacturer's protocol. First strand cDNA was synthesized using PrimeScript™ RT Reagent Kit with gDNA Eraser (TaKaRa Bio Inc.). Candidate gene expression levels were measured using an RT-PCR thermocycler (StepOne™ Life Technologies) with the following specific primers: IL-1*β*, forward: 5′-GTGGCAGCTACCTATGTCTTGC-3′, reverse: 5′-CCACTTGTTGGCTTATGTTCTGT-3′; IL-6, forward: 5′-TGGAGTTCCGTTTCTACCTGG-3′, reverse: 5′-GGTCCTTAGCCACTCCTTCTGT-3′; and TNF-*α*, forward: 5′-CACCACGCTCTTCTGTCTACTG-3′, reverse: 5′-GCTACGGGCTTGTCACTCG-3′. The mRNA level for each target gene was calculated using the Delta-Delta-CT method and normalized to the *β*-actin mRNA level from the same sample. The primers for *β*-actin were as follows: forward: 5′-CGTTGACATCCGTAAAGACCTC-3′, reverse: 5′-TAGGAGCCAGGGCAGTAATCT-3′.

### 2.10. Statistical Analysis

Continuous variables are expressed as the mean ± SD and were analyzed by one-way ANOVA. All data were analyzed using GraphPad Prism version 7.0 software (GraphPad Software, Inc. San Diego, CA), and two-tailed *p* ≤ 0.05 was considered significant.

## 3. Results

### 3.1. VNS Significantly Alleviated SMI/R Injury

**Figure 2:**
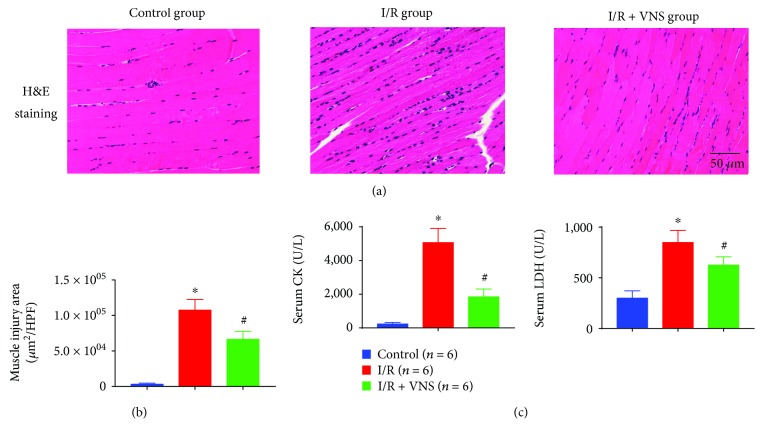
VNS alleviates skeletal muscle injury. (a) Control group: normal skeletal muscle cell shape and arrangement; I/R group: skeletal muscle cell degeneration and necrosis, wide range of inflammatory cell accumulation; I/R+VNS group: mixed normal and pathological skeletal muscle cells, little inflammatory cell infiltration. (b) The muscle injury area in the three groups. (c) The serum concentration of CK and LDH. ^∗^*p* < 0.05 vs. control group; ^#^*p* < 0.05 vs. I/R group; HPF: high-power field; CK: creatine kinase; LDH: lactic dehydrogenase.

As shown in Figure 2(a), skeletal muscle tissues in the I/R group showed muscle cell degeneration, necrosis, sarcoplasmic dissolution, and neutrophil infiltration. The extent of these changes was alleviated in the I/R+VNS group.  Figure 2(b) shows that the gastrocnemius muscle injury area in the high-power field (HPF) was highest in the I/R group, significantly lower in the I/R+VNS group, and lowest in the control group.

Figure 2(c) shows that the CK and LDH levels were significantly higher in the I/R group than in the control group (CK, 5060.31 ± 847.02 vs. 233.13 ± 98.01 U/L, *p* < 0.05; LDH, 847.96 ± 120.20 vs. 298.50 ± 73.10 U/L, *p* < 0.05). The CK and LDH levels were significantly attenuated by the VNS treatment (CK, 1849.44 ± 456.94 vs. 5060.31 ± 847.02 U/L, *p* < 0.05; LDH, 625.65 ± 81.51 vs. 847.96 ± 120.20 U/L, *p* < 0.05).

**Figure 3:**
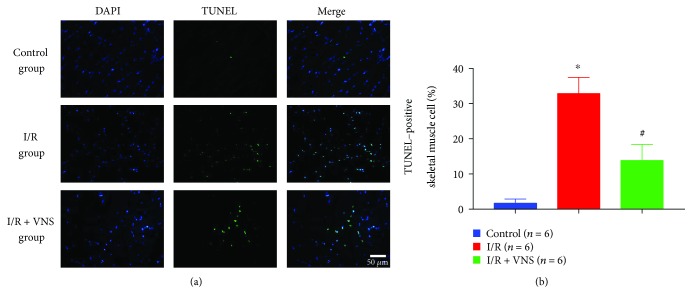
Effect of VNS on skeletal muscle cell apoptosis. (a) Representative images showing immunofluorescence staining for DAPI (blue) and TUNEL (green) in skeletal muscle cell nuclei from the control, I/R, and I/R+VNS groups. (b) Quantification of skeletal muscle cell apoptosis. ^∗^*p* < 0.05 vs. control group; ^#^*p* < 0.05 vs. I/R group.

TUNEL staining was used to detect skeletal muscle cell apoptosis among the three groups. The percentage of TUNEL-positive cells was significantly increased in the I/R group compared with the control group (32.83 ± 4.62% vs. 1.67 ± 1.21%, *p* < 0.05). In the I/R+VNS group, the percentage of TUNEL-positive cells was significantly decreased compared with that in the I/R group (13.83 ± 2.59% vs. 32.83 ± 4.62%, *p* < 0.05) (see Figure 3).

### 3.2. VNS Significantly Mitigated the Inflammatory Response in the Skeletal Muscle after I/R

**Figure 4:**
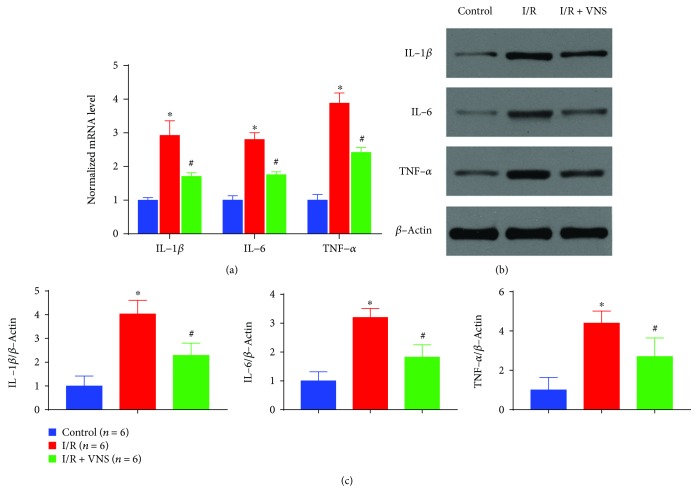
VNS mitigates inflammation in SMI/R injury. (a) Relative mRNA levels of IL-1*β*, IL-6, and TNF-*α* in the control, I/R, and I/R+VNS groups. (b, c) Representative blots and relative protein expression of IL-1*β*, IL-6, and TNF-*α* among different groups. ^∗^*p* < 0.05 vs. control group; ^#^*p* < 0.05 vs. I/R group. IL-1*β*: interleukin-1*β*; IL-6: interleukin-6; TNF-*α*: tumor necrosis factor-*α*.

As shown in Figure 4, the mRNA and protein expression levels of proinflammatory factors IL-1*β*, IL-6, and TNF-*α* were markedly increased in the I/R group compared with the control group (mRNA expression: IL-1*β*, 2.92 ± 0.44 vs. 1.00 ± 0.08; IL-6, 2.80 ± 0.20 vs. 1.00 ± 0.13; and TNF-*α*, 3.88 ± 0.31 vs. 1.00 ± 0.17, *p* < 0.05 for each. Protein expression: IL-1*β*, 4.03 ± 0.58 vs. 1.00 ± 0.42; IL-6, 3.20 ± 0.32 vs. 1.00 ± 0.31; and TNF-*α*, 4.40 ± 0.62 vs. 1.00 ± 0.6, *p* < 0.05 for each). VNS significantly mitigated the increased expression of levels of inflammatory markers (mRNA expression: IL-1*β*, 1.71 ± 0.11 vs. 2.92 ± 0.44; IL-6, 1.76 ± 0.09 vs. 2.80 ± 0.20; and TNF-*α*, 2.42 ± 0.15 vs. 3.88 ± 0.31, *p* < 0.05 for each. Protein expression: IL-1*β*, 2.29 ± 0.51 vs. 4.03 ± 0.58; IL-6, 1.83 ± 0.43 vs. 3.20 ± 0.32; and TNF-*α*, 2.70 ± 0.0.74 vs. 4.40 ± 0.62, *p* < 0.05 for each).

### 3.3. VNS Significantly Attenuated Oxidative Stress in Skeletal Muscle after I/R

**Figure 5:**
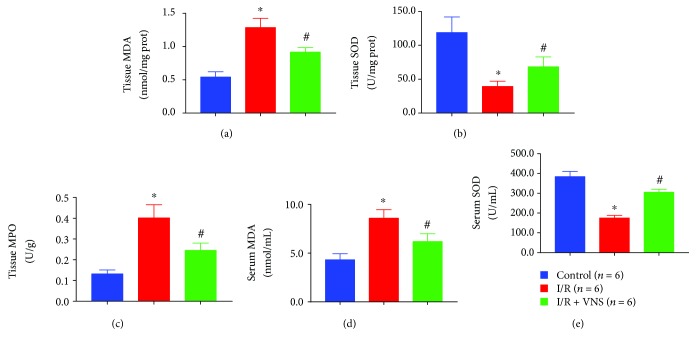
VNS attenuates oxidative stress in SMI/R injury. (a-c) The effect of VNS on the MDA, SOD, and MPO levels in skeletal muscle tissues. (d-e) The effect of VNS on the MDA and SOD levels in serum. ^∗^*p* < 0.05 vs. control group; ^#^*p* < 0.05 vs. I/R group. MDA: myeloperoxidase; SOD: superoxide dismutase; MPO: malondialdehyde.

The MPO activity in skeletal muscle tissues was used to indicate the neutrophilic infiltration severity. I/R injury significantly increased the MPO activity compared with the sham operation (0.40 ± 0.06 vs. 0.13 ± 0.02 U/g, *p* < 0.05). In contrast, VNS significantly alleviated the increase in MPO activity after I/R (0.25 ± 0.04 vs. 0.40 ± 0.06 U/g, *p* < 0.05) (see Figure 5(c)). The changes in the MDA and SOD concentrations in skeletal muscle tissues are shown in Figures 5(a)-5(b). As a biomarker of oxidative stress, the MDA level was significantly increased in tissues after I/R (1.29 ± 0.14 vs. 0.54 ± 0.08 nmol/mg protein, *p* < 0.05). In contrast, as a biomarker of antioxidant activity, the SOD was significantly decreased in tissues after I/R (39.26 ± 7.85 vs. 118.85 ± 23.00 U/mg protein, *p* < 0.05). However, VNS during I/R markedly mitigated these changes (MDA, 0.92 ± 0.07 vs. 1.29 ± 0.14 nmol/mg protein; SOD, 68.31 ± 14.46 vs. 39.26 ± 7.85 U/mg protein, *p* < 0.05). Similar variation trends were observed for the serum MDA and SOD levels (see Figures 5(d)-5(e)). Compared to the control group, the I/R group showed significantly different serum MDA and SOD levels (MDA, 8.58 ± 0.88 vs. 4.32 ± 0.62 nmol/mL; SOD, 175.14 ± 13.10 vs. 383.93 ± 26.28 U/mL, *p* < 0.05 for both), while VNS markedly attenuated these changes (MDA, 6.19 ± 0.80 vs. 8.58 ± 0.88 nmol/mL; SOD, 305.37 ± 14.76 vs. 175.14 ± 13.10 U/mL, *p* < 0.05 for both).

### 3.4. VNS Significantly Protected Vascular Endothelial Function in the Skeletal Muscle after I/R

**Figure 6:**
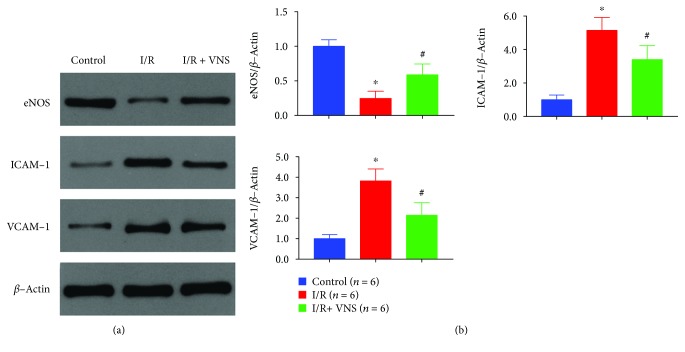
VNS protects vascular endothelial function. (a) Representative examples of eNOS, ICAM-1, and VCAM-1 expression in different groups. (b) Relative expression levels are shown as the ratio between the target proteins and *β*-actin expression levels. ^∗^*p* < 0.05 vs. control group; ^#^*p* < 0.05 vs. I/R group. eNOS: endothelial nitric oxide synthase; ICAM-1: intercellular cell adhesion molecule-1; VCAM-1: vascular cell adhesion molecule-1.

Endothelial function was evaluated by the expression of eNOS, ICAM-1, and VCAM-1.  Figure 6(b) shows that I/R significantly decreased the expression level of eNOS and increased the expression levels of ICAM-1 and VCAM-1, while VNS exerted a protective effect on endothelial function and relieved the above changes. The relative expression of eNOS, ICAM-1, and VCAM-1 in the control and I/R groups were 1.00 ± 0.09 vs. 0.25 ± 0.10, 1.00 ± 0.28 vs. 5.15 ± 0.77, and 1.00 ± 0.20 vs. 3.82 ± 0.59, respectively, with *p* < 0.05 for all. VNS significantly relieved the changes described above (0.59 ± 0.16 vs. 0.25 ± 0.10, 3.40 ± 0.83 vs. 5.15 ± 0.77, and 2.14 ± 0.61 vs. 3.82 ± 0.59 for eNOS, ICAM-1, and VCAM-1, respectively, *p* < 0.05 for all).

## 4. Discussion

In the present study, we provide novel evidence that VNS during SMI/R injury could ameliorate skeletal muscle injury, as shown by alleviated cellular apoptosis, degeneration and inflammatory cell infiltration, and reduced serum CK and LDH levels compared to sham VNS. The underlying mechanisms of this protective effect involve inhibiting excessive inflammation and oxidative stress and protecting endothelial function. To the best of our knowledge, this is the first study to apply VNS to treat SMI/R injury.

It is well known that the innervation of the autonomic nervous system plays a vital role during organic I/R injury, including SMI/R injury. Sympathetic nerves are distributed in the adventitia of arteries in the skeletal muscle [14]. Increasing the sympathetic tone will lead to enhanced noradrenaline release, which results in vasoconstriction via direct activation of the *α*-receptors and inhibition of vasodilating neuropeptides. Ischemia and hypoxia are common sympathoexcitatory stresses. This sympathetic vasoconstrictive effect might further exaggerate SMI/R injury [15]. Povlsen and Sirsjo have reported that treatment with guanethidine, a sympathetic nerve blocker, during reperfusion in SMI/R leads to a better prognosis [16]. Increased vagal tone might offset the sympathetic vasoconstrictive effect and protect organs from I/R injury. There are also emerging studies of this potential therapy for I/R injury. In a rat model, Jiang et al. reported that VNS treatment during cerebral I/R significantly reduced I/R injury [17]. The heart muscle is histologically similar to the skeletal muscle, and in a cardiac I/R model, VNS significantly reduced reperfusion arrhythmias and infarct size [8]. Our previous study also showed an analogous protective effect of VNS against myocardial I/R injury [18]. Such effects suggest that VNS might be a potential approach for treating SMI/R. The results of the present study are consistent with those of previous studies and demonstrate that VNS can attenuate SMI/R injury.

Currently, it is recognized that the pathophysiological mechanisms of I/R injury in the skeletal muscle include inflammation, oxidative stress, vascular endothelial damage, calcium overload, and mitochondria damage [19–22]. Corrick et al. reported that the administration of dexamethasone, a classic anti-inflammatory drug, at the onset of reperfusion ameliorated the structural and functional damage in the skeletal muscle [23], indicating the important role of inflammation in SMI/R injury. Acetylcholine (Ach) is an anti-inflammatory substance through cholinergic anti-inflammatory pathways [24]. VNS has been demonstrated to stimulate the release of Ach and might be a potential anti-inflammatory treatment in different diseases. Jonge et al. have reported that VNS could attenuate macrophage activation and suppress inflammation [25]. Similar results have been reported by Koopman et al., indicating that VNS could inhibit proinflammatory cytokine production and attenuate inflammatory disease [26]. Several researchers have applied the vagal anti-inflammatory effect in I/R injury and achieved excellent effects. Inoue et al. reported that VNS significantly reduced the expression of proinflammatory cytokines in a renal I/R rat model [7]. Similarly, our data indicate that VNS significantly reduced the inflammatory cytokine levels compared with sham VNS.

Reperfusion induced oxidative stress can promote skeletal muscle cell apoptosis [27]. It is known that inhibiting oxidative stress can effectively alleviate I/R injury. One study has shown that VNS can reduced oxidative stress in a cerebral I/R rat model [17]. Our previous study indicated that VNS markedly reduced reactive oxygen species (ROS) production in a myocardial I/R canine model [18]. These data show that VNS could prevent I/R injury via an antioxidative stress effect. MPO is involved in the generation of ROS. The balance between prooxidant biomarkers (MDA) and antioxidant biomarkers (SOD) represents the activity of oxidant stress [28]. In the present study, our data suggest that VNS significantly decreased tissue MPO activity, reduced the concentration of MDA, and increased SOD activity in both tissue and serum. Therefore, we suggest that antioxidative activity may be one of potential mechanisms underlying the protective effect of VNS against SMI/R injury.

Vascular endothelial dysfunction is another mechanism involved in SMI/R injury [29]. Endothelial activation, which is defined by increased expression of cell surface adhesion molecules such as ICAM-1 and VCAM-1, was the main manifestation of vascular endothelial dysfunction [30]. The inflammatory response could activate the vascular endothelium, promote the production of proinflammatory cytokines (such as IL-6 and TNF-*α*), and increase the expression of ICAM-1 and VCAM-1. A previous study has shown that cholinergic agonists could suppress endothelial cell activation, as confirmed by decreased ICAM-1 expression [31]. Our results show that VNS relieved the increased expression of ICAM-1 and VCAM-1 induced by SMI/R injury. Nitric oxide (NO) is a bioactive molecule that helps dilate the blood vessels. It has been proven that NO could inhibit inflammatory cell adhesion and limit endothelial activation [32]. The expression of eNOS is essential for the production of NO and integrity of the vascular endothelium [33]. Yoshizumi et al. reported inhibiting eNOS expression could lead to endothelial dysfunction in human umbilical vein endothelial cells [34]. Previous studies have shown that increased eNOS expression leads to improvement of the endothelial function, which could improve the prognosis of SMI/R injury [11]. Li et al. reported that chronic VNS increased the expression of eNOS in ovariectomized rats [35]. In the present study, VNS significantly increased the expression of eNOS compared with sham VNS. Together with the downregulated expression of ICAM-1 and VCAM-1 and the increased eNOS expression results, our data indicated that VNS could protect the skeletal muscle from I/R injury by preserving endothelial function.

**Figure 7:**
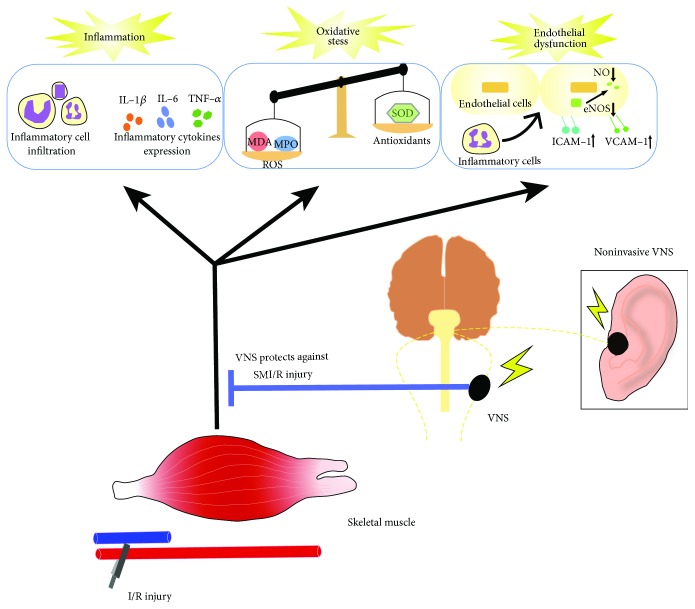
Schematic diagram depicting the protective effect and potential mechanisms of VNS on SMI/R injury. Noninvasive VNS may become a safe and acceptable technology for treating SMI/R patients.

SMI/R injury is a common clinical condition. Until now, effective drug interventions to address this pathological state have been limited. The present study provides evidence that VNS could markedly reduce skeletal muscle tissue injury and cell apoptosis induced by I/R. Recently, stimulating the auricular branch of the vagus nerve has been proven to be safe and effective for achieving a similar effect as cervical VNS, which might overcome the shortcomings of conventional VNS [10, 36]. With further research to validate its safety and practicability, noninvasive VNS might become a novel technology to treat SMI/R injury in patients (see Figure 7).

### 4.1. Study Limitations

First, different stimulation sites and parameters have been shown to exert distinct therapeutic effects. In this study, we only verified the current stimulation parameters in left-side VNS. Further studies will aim to contrast bilateral VNS and explore the best stimulation parameters. Second, although we revealed several potential mechanisms by which VNS protects against SMI/R injury, the exact mechanism remains to be explored. Third, we only investigated the acute impact of VNS on SMI/R injury. Long-term effects should be verified in future studies. Fourth, we only measured the expression of eNOS as a parameter to reflect the endothelial function. The eNOS activity and NO production can be better to reflect the endothelial function. The effect of VNS on these parameters will be investigated in our further studies.

## 5. Conclusions

In conclusion, our data suggest that VNS could play a protective role in I/R-induced skeletal muscle injury. Its potential mechanisms may involve suppressing excessive inflammation, alleviating oxidative stress, and preserving vascular endothelial function. Although further studies are needed to validate its safety and practicability, VNS might provide a novel treatment for patients with SMI/R injury.

## Data Availability

The data used to support the findings of this study are available from the corresponding author upon request.

## References

[1] Walker P. M. (1991). Ischemia/reperfusion injury in skeletal muscle. *Annals of Vascular Surgery*.

[2] Gehrig S. M., Lynch G. S. (2011). Emerging drugs for treating skeletal muscle injury and promoting muscle repair. *Expert Opinion on Emerging Drugs*.

[3] Blaisdell F. W. (2002). The pathophysiology of skeletal muscle ischemia and the reperfusion syndrome: a review. *Cardiovascular Surgery*.

[4] Borovikova L. V., Ivanova S., Zhang M. (2000). Vagus nerve stimulation attenuates the systemic inflammatory response to endotoxin. *Nature*.

[5] Wang H., Yu M., Ochani M. (2003). Nicotinic acetylcholine receptor alpha7 subunit is an essential regulator of inflammation. *Nature*.

[6] Bonaz B., Picq C., Sinniger V., Mayol J. F., Clarencon D. (2013). Vagus nerve stimulation: from epilepsy to the cholinergic anti-inflammatory pathway. *Neurogastroenterology and Motility*.

[7] Inoue T., Abe C., Sung S. S. J. (2016). Vagus nerve stimulation mediates protection from kidney ischemia-reperfusion injury through *α*7nAChR^+^ splenocytes. *The Journal of Clinical Investigation*.

[8] Shinlapawittayatorn K., Chinda K., Palee S. (2013). Low-amplitude, left vagus nerve stimulation significantly attenuates ventricular dysfunction and infarct size through prevention of mitochondrial dysfunction during acute ischemia-reperfusion injury. *Heart Rhythm*.

[9] Jiang Y., Li L., Liu B., Zhang Y., Chen Q., Li C. (2015). PPAR*γ* upregulation induced by vagus nerve stimulation exerts anti-inflammatory effect in cerebral ischemia/reperfusion rats. *Medical Science Monitor*.

[10] Yu L., Huang B., Po S. S. (2017). Low-level tragus stimulation for the treatment of ischemia and reperfusion injury in patients with ST-segment elevation myocardial infarction: a proof-of-concept study. *JACC: Cardiovascular Interventions*.

[11] Hallström S., Gasser H., Neumayer C. (2002). S-nitroso human serum albumin treatment reduces ischemia/reperfusion injury in skeletal muscle via nitric oxide release. *Circulation*.

[12] Liu A. F., Zhao F. B., Wang J. (2016). Effects of vagus nerve stimulation on cognitive functioning in rats with cerebral ischemia reperfusion. *Journal of Translational Medicine*.

[13] Yin T. C., Wu R. W., Sheu J. J. (2018). Combined therapy with extracorporeal shock wave and adipose-derived mesenchymal stem cells remarkably improved acute ischemia-reperfusion injury of quadriceps muscle. *Oxidative Medicine and Cellular Longevity*.

[14] Shoemaker J. K., Badrov M. B., Al-Khazraji B. K., Jackson D. N. (2015). Neural control of vascular function in skeletal muscle. *Comprehensive Physiology*.

[15] Hagström-Toft E., Enoksson S., Moberg E., Bolinder J., Arner P. (1998). *β*-Adrenergic regulation of lipolysis and blood flow in human skeletal muscle in vivo. *American Journal of Physiology-Endocrinology and Metabolism*.

[16] Povlsen B., Sirsjo A. (1999). Sympathetic block significantly improves reperfusion in skeletal muscle following prolonged use of tourniquet. *Journal of Hand Surgery*.

[17] Jiang Y., Li L., Tan X., Liu B., Zhang Y., Li C. (2015). miR-210 mediates vagus nerve stimulation-induced antioxidant stress and anti-apoptosis reactions following cerebral ischemia/reperfusion injury in rats. *Journal of Neurochemistry*.

[18] Chen M., Zhou X., Yu L. (2016). Low-level vagus nerve stimulation attenuates myocardial ischemic reperfusion injury by antioxidative stress and antiapoptosis reactions in canines. *Journal of Cardiovascular Electrophysiology*.

[19] Gillani S., Cao J., Suzuki T., Hak D. J. (2012). The effect of ischemia reperfusion injury on skeletal muscle. *Injury*.

[20] Carden D. L., Granger D. N. (2000). Pathophysiology of ischaemia-reperfusion injury. *The Journal of Pathology*.

[21] Odeh M. (1991). The role of reperfusion-induced injury in the pathogenesis of the crush syndrome. *The New England Journal of Medicine*.

[22] Eltzschig H. K., Eckle T. (2011). Ischemia and reperfusion--from mechanism to translation. *Nature Medicine*.

[23] Corrick R. M., Tu H., Zhang D. (2018). Dexamethasone protects against tourniquet-induced acute ischemia-reperfusion injury in mouse hindlimb. *Frontiers in Physiology*.

[24] Rosas-Ballina M., Olofsson P. S., Ochani M. (2011). Acetylcholine-synthesizing T cells relay neural signals in a vagus nerve circuit. *Science*.

[25] de Jonge W. J., van der Zanden E. P., The F. O. (2005). Stimulation of the vagus nerve attenuates macrophage activation by activating the Jak2-STAT3 signaling pathway. *Nature Immunology*.

[26] Koopman F. A., Chavan S. S., Miljko S. (2016). Vagus nerve stimulation inhibits cytokine production and attenuates disease severity in rheumatoid arthritis. *Proceedings of the National Academy of Sciences of the United States of America*.

[27] Sies H. (2015). Oxidative stress: a concept in redox biology and medicine. *Redox Biology*.

[28] Frijhoff J., Winyard P. G., Zarkovic N. (2015). Clinical relevance of biomarkers of oxidative stress. *Antioxidants & Redox Signaling*.

[29] Sternbergh W. C., Adelman B. (1992). The temporal relationship between endothelial cell dysfunction and skeletal muscle damage after ischemia and reperfusion. *Journal of Vascular Surgery*.

[30] Liao J. K. (2013). Linking endothelial dysfunction with endothelial cell activation. *The Journal of Clinical Investigation*.

[31] Chatterjee P. K., Al-Abed Y., Sherry B., Metz C. N. (2009). Cholinergic agonists regulate JAK2/STAT3 signaling to suppress endothelial cell activation. *American Journal of Physiology Cell Physiology*.

[32] De Caterina R., Libby P., Peng H. B. (1995). Nitric oxide decreases cytokine-induced endothelial activation. Nitric oxide selectively reduces endothelial expression of adhesion molecules and proinflammatory cytokines. *The Journal of Clinical Investigation*.

[33] Siragusa M., Fleming I. (2016). The eNOS signalosome and its link to endothelial dysfunction. *Pflügers Archiv*.

[34] Yoshizumi M., Perrella M. A., Burnett J. C., Lee M. E. (1993). Tumor necrosis factor downregulates an endothelial nitric oxide synthase mRNA by shortening its half-life. *Circulation Research*.

[35] Li P., Liu H., Sun P. (2016). Chronic vagus nerve stimulation attenuates vascular endothelial impairments and reduces the inflammatory profile via inhibition of the NF-*κ*B signaling pathway in ovariectomized rats. *Experimental Gerontology*.

[36] Fang J., Rong P., Hong Y. (2016). Transcutaneous vagus nerve stimulation modulates default mode network in major depressive disorder. *Biological Psychiatry*.

